# Metastatic uveal melanoma showing durable response to anti-CTLA-4 and anti-PD-1 combination therapy after experiencing progression on anti-PD-1 therapy alone

**DOI:** 10.1186/s40425-018-0322-1

**Published:** 2018-02-12

**Authors:** Muhammad Zubair Afzal, Rodwell Mabaera, Keisuke Shirai

**Affiliations:** 1 Department of Hospital Medicine, Dartmouth-Hitchcock Medical Center, One Medical Center Dr., Lebanon, NH 03756 USA; 2 Hematology/Oncology, Norris cotton Cancer Center, One Medical Center Dr., Lebanon, NH 03756 USA

## Abstract

**Background:**

Uveal melanoma accounts for 85% of the ocular melanomas and has an increased risk of hematogenous spread, most commonly to the liver. After curative intent therapy like surgery and radiation, fifty percent of patients present with distant metastasis. Metastatic uveal melanoma (MUM) does not harbor typically targetable mutations, e.g., BRAF as in cutaneous melanoma. As a result, there is no proven therapy for MUM. Various chemotherapy and immunotherapy regimens have been tried and only partial response (PR) is the best that has been achieved in most of the cases. Here, we present a case of MUM treated with combination immune checkpoint therapy (ipilimumab and nivolumab) following the progression with single-agent nivolumab and demonstrating a durable response without recurrence more than 22 months from the last treatment.

**Case Presentation:**

A 72-year-old Caucasian man presented with ciliary body melanoma of the left eye and underwent curative-intent enucleation but six months later developed diffuse hepatic metastases. He initially was treated with nivolumab 3 mg/kg every two weeks for four cycles but restaging scan showed a significant progression of the disease with increasing LDH. With the FDA approval for the combination of nivolumab 1mg/kg with Ipilimumab 3 mg/kg every three weeks for metastatic melanoma, this combination was given for four cycles with continuous rise in LDH to 993 unit/L (110-220 unit/L) until finishing cycle four of the treatment. Three weeks later, maintainence nivolumab 3mg/kg was initiated but two weeks later, he developed grade 4 liver toxicity with ALT 1565 unit/L (0-55 unit/L). A presumptive diagnosis of autoimmune hepatitis was made, nivolumab was stopped and oral prednisone 1mg/kg was started with quick resolution of elevated transaminases. Restaging abdominal MRI one month after the first and last dose of maintenance nivolumab showed PR and continuous shrinkage of the metastatic lesions with no hypermetabolic activity even on PET/CT. He is 22 months' post-treatment and continues to do well without any evidence of active disease.

**Conclusion:**

Although, limited response has been shown to single agent immune checkpoint inhibitors and chemotherapy, our patient showed durable response with anti-CTLA-4 and anti-PD-1 combination therapy in MUM.

## Background

Uveal melanoma arises from the melanocytes in the iris, ciliary body, or choroid [1]. Although the most common primary intraocular malignancy in adults (85% of all ocular melanomas), it is very rare with an incidence of about five per one million persons each year [1, 2]. Surgical enucleation and advances in radiotherapy techniques have improved local control, however up to 50% of the patients’ relapse after a curative-intent local therapy [2–4], and eventually require systemic treatments. Due to lack of draining lymphatics, uveal melanoma has early hematogenous dissemination [5], with 80–90% of patients with metastatic uveal melanoma (MUM) presenting with liver as the first site of disease involvement. Lungs are involved in 29%, and bone is involved in 17% of the cases [6].

Historically, MUM has been considered to have the worse prognosis and poorer response to chemotherapy partly due to a rarity of the diagnosis and/or exclusion of MUM patients from large randomized clinical trials [2, 7]. A systematic review that included 841 patients from 40 different reports, mostly nonrandomized phase II studies, showed an overall response rate (ORR) of only 4.6% with 22 studies showing no response in any patients [8]. There was a tendency for higher response rates in studies that used chemo-immunotherapy regimens. Notably, chemotherapy alone did not have an impact on overall survival (OS).

Unlike cutaneous melanoma, which has benefited from therapies targeting mutated Braf, uveal melanoma does not harbor these mutations. Based on one study selumetinib, a MEK 1/2 inhibitor, was considered a promising agent in the treatment of MUM and granted orphan status by FDA for this indication based on significantly increased ORR (14 vs. 0%) in combination with temozolomide compared to temozolomide alone [9, 10]. The same study also demonstrated improved median PFS of 15.9 weeks from single-agent selumetinib compared with 7 weeks from chemotherapy (HR = 0.46; 95% CL, 0.30–0.71; *P* < 0.001), however, no improvement in OS was observed in this study (HR = 0.66; 95% CI, 0.41–1.06; *P* = 0.09) [10].

Due to the marginal benefits from selumetinib compared to chemotherapy and the approval of immune checkpoint inhibitors in 2011, several case reports and series have investigated ipilimumab for treatment of MUM. These studies highlight the low ORR (≤ 5% for MUM in contrast to ~ 20% for cutaneous melanoma) [8]. In the three expanded access programs that evaluated clinical activity of ipilimumab in MUM, only two of 56 patients (3.6%) experienced a partial response (PR) while twelve patients showed disease stabilization [11–13]. Luke J. et al. described a PR in only one out of 39 (2.5%) patients with MUM treated with ipilimumab and stable disease (SD) in another six (15.38%) patients at last follow-up [14]. One case series reported no objective responses in 21 patients treated with either nivolumab or pembrolizumab in previously treated MUM patients with only six of fourteen (42.85%) patients experiencing SD as the best response [15]. In another study of 56 patients who have received prior therapy, three patients had an objective response to ipilimumab and eight patients had SD as their best response [16]. These results emphasize the need for prospective trials to evaluate the role of these agents in the first line treatment of MUM, and question that is limited by the ability to recruit a large enough cohort of patients. Recently, a systematic review by Komastsubara et al. specifies the role of immunotherapy for the treatment of MUM and summarizes multiple cases treated with single anti-CTLA-4 or anti-PD-1/PD-L1 antibodies. They conclude that the success achieved by these agents in metastatic cutaneous melanoma has not been reciprocated in MUM patients to the same extent. Currently there are clinical trials with a combination of anti-CTLA-4 antibody and anti-PD-1 antibody under recruitment [17]. A clinical trial (clinical-trials.gov NCT01585194) is currently recruiting patients with Uveal melanoma; this is a phase II trial using nivolumab in combination with ipilimumab. Another trial (*clinical-trials.gov* NCT02626962) is aimed at treatment of previously treated MUM patients with nivolumab in combination with ipilimumab. This trial, however is not recruiting patients yet. To this point, we present a case of MUM treated with combination immune checkpoint therapy (Anti-PD-1 and Anti-CTLA-4) following the failure of single-agent nivolumab and demonstrate a durable response months after receiving treatment with nivolumab and ipilimumab combination.

## Case presentation

Our patient is a 72-year-old man with a history of Sweet’s syndrome, hypertension, hyperlipidemia, basal cell carcinoma and psoriasis. He presented with acute painless vision loss described as a rapidly progressing “curtain” over his left eye in December 2014. There was no history of trauma or other antecedent events to have caused retinal detachment. Emergent examination of the eye revealed an approximately 2-cm mass lesion and ultrasound confirmed a 1.2-cm dome-shaped lesion involving the ciliary body. Laboratory evaluations including complete blood counts, chemistries, and hepatic function tests were normal at that time. Brain MRI confirmed a left globe lesion tracking along the retina, but no evidence of other intracranial lesions and positron emission tomography/computed tomography (PET/CT) did not show any evidence of metastatic disease.

He underwent a curative-intent enucleation two months later with pathology confirming left ciliary body melanoma. Primary pathology showed ciliochoroidal malignant melanoma with no extra-scleral extension. The tumor had zones of necrosis and numerous areas with epithelioid and spindle melanoma cells. There were areas of necrosis within the tumor but no evidence of extra-scleral extension.

**Fig. 1:**
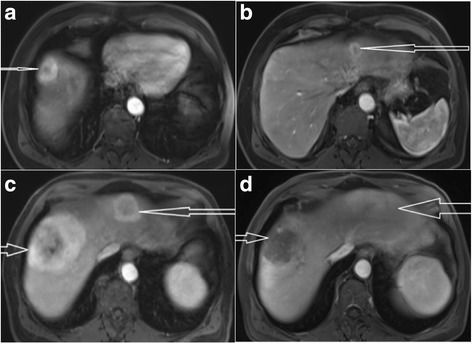
MRI abdomen T2-hyperintense signal. **a** & **b** showing multiple metastatic lesions to the liver before the initiation of immunotherapy. **c** showing progression of the disease after 4 cycles of nivolumab. The largest lesion was 5.5 cm in the right hepatic lobe. **d** showing a mixed response after 4 cycles on ipilimumab/nivolumab

Unfortunately, his initial surveillance PET/CT scan six months after enucleation revealed diffuse liver metastases. Laboratory evaluations remained normal including his lactate dehydrogenase (LDH). MRI of the liver confirmed numerous enhancing lesions, with the largest measuring 3.8 cm in the anterior right lobe [Fig. 1a and b].

Liver biopsy confirmed this to be metastatic melanoma consistent with an ocular primary. Molecular profiling did not reveal actionable mutations in c-kit, Braf, or Ras, but did show a mutation in GNA11 (codon 626 A > T). He was treated with nivolumab 3 mg/kg every two weeks and completed four cycles prior to obtaining a restaging MRI. Unfortunately, this showed the progression of disease in the liver with the largest right lobe lesion now measuring 5.5 cm [Fig. 1c]. The patient also reported increasing vague abdominal fullness, intermittent nausea and his LDH was noted to be rising (333 U/L, upper limit of normal 220).

**Fig. 2:**
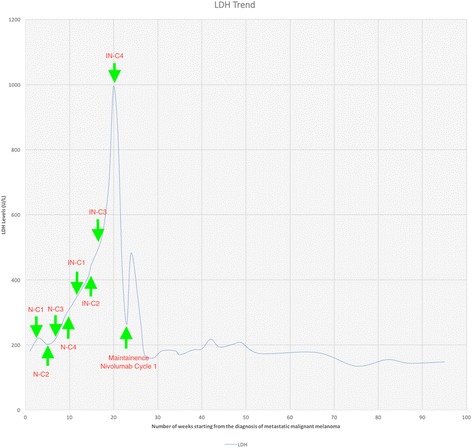
Showing the LDH trend starting from the diagnosis of the metastatic malignant melanoma. LDH started rising during 4 cycles of nivolumab therapy and the patient had LDH of 333 U/L at the time of C1 of nivolumab/ipilimumab. LDH reaching its peak by the time patient received C4 of nivolumab/ipilimumab. After 4 cycles of ipilimumab/nivolumab, the LDH started to decrease, falling from peak of 993 U/L to 420 and then continue to fall. 2nd peak (LDH of 483) was observed 2 weeks after the start of Nivolumab C1 before continuing the downtrend. [C = Cycle, N = Nivolumab, IN = Ipilimumab/Nivolumab]

Later that month, following FDA approval, the patient was started on nivolumab 1 mg/kg with ipilimumab 3 mg/kg every three weeks. A restaging abdominal CT scan after two cycles of treatment showed overall stable disease in size and, his increasing abdominal symptoms were felt to be related to the liver disease as he continued to experience rise in his LDH (now 514); Following completion of therapy cycle four, his abdominal symptoms started to improve, and his LDH started to decrease falling from a peak of 993 to 420 U/L over three weeks [Fig. 2]. A restaging MRI, three weeks after cycle number four showed mixed response with some signal changes consistent with treatment effect but other lesions were concerning for progression [Fig. 1d].

**Fig. 3:**
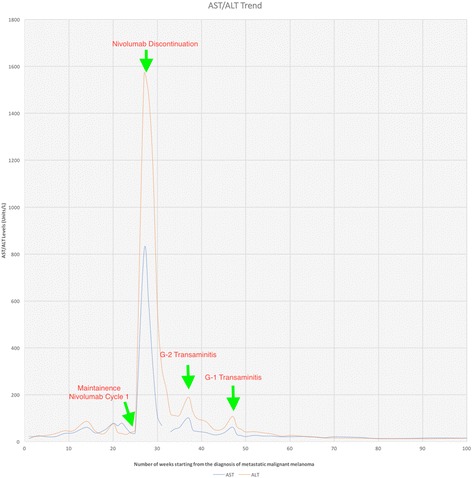
Showing stable AST/ALT trend since diagnosis of malignant melanoma before peaking 2 weeks after starting nivolumab maintenance therapy. AST peaked at 811 and ALT peaked at 1565 U/L. Transaminitis improved after initiation of prednisone 1 mg/kg and stopping nivolumab. Patient experienced another episode of grade 2 transaminitis and his prednisone was increased back to 20 mg per day before tapering again. Patient had another episode of grade 1 transaminitis resulting in small adjustment of prednisone. [G = Grade]

He was thus started on maintenance nivolumab three weeks from cycle four of ipilimumab/nivolumab. Two weeks later when he presented for cycle two maintenance nivolumab he was found to have mild fatigue with grade 4 transaminitis with AST 811 (normal < 39), ALT 1565 (normal < 55) and a normal bilirubin. He was diagnosed with presumed autoimmune hepatitis and started on oral prednisone 1 mg/kg while subsequent nivolumab was held. He had a quick response to steroids, with his transaminitis improved down to grade 1, and was tapered to 15 mg/day over one month followed by 5 mg/day for an additional month [Fig. 3]. Restaging MRI done around two months from cycle one, the first and last dose of maintenance nivolumab, showed the decreased size of the numerous hepatic metastases without evidence of new metastatic disease. Surveillance PET/CT scans 4, 11 and 18 months from the last nivolumab showed no hypermetabolic activity without evidence of other metastatic disease. Surveillance abdominal MRI scans 2,8,12 and 15 months from the last nivolumab showed continuous shrinkage of liver lesions without enhancement. Latest surveillance abdominal MRI scan 22 months from the last dose of nivolumab showed further interval retraction of the liver lesions.

Three months after the diagnosis of grade 4 transaminitis, his prednisone was increased to 20 mg a day for recurrent grade 2 transaminitis but was able to taper quickly to 5 mg/day. Currently, after 22 months from the diagnosis of grade 4 transaminitis, he is maintained on 2.5 mg prednisone daily for fatigue. Patient has been off any therapy for more than 22 months now and is on low dose prednisone 2.5 mg/ day for 14 months now. Most recent AST/ALT values were 15/14 units/L respectively.

## Discussion

Uveal melanoma differs from the cutaneous melanoma in characteristics and prognosis. Monosomy 3, 1p loss, 1 q gain, 6 p gain, 8 p loss and 8 q gain are the most common chromosomal abnormalities seen in uveal melanoma. Monosomy 3 is associated with metastasis in approximately 50% of the cases and is associated with worse prognosis [18]. Chromosome 6q loss, 8q gain or 8p loss are associated with the poor prognosis [7]. Furthermore, greater than 80% of uveal melanomas possess oncogenic mutations in G-protein-α subunits associated genes *GNAQ* or *GNA11* as found in our case [19]. These mutations are also associated with poor prognosis. Some other factors related to poor prognosis are the older age at presentation, male gender, short metastasis free interval, extraocular extension ciliary body involvement which were all present in our patient [7, 20].

More than 50% of the patients treated with curative intent for localized uveal melanoma present with metastatic disease. This is associated with poor prognosis and predictably poor responses to conventional therapies [7]. Recent case reports and studies have also shown that the response rates to single-agent immune checkpoint blockade are very low with only a handful of patients experiencing objective responses to date [11–16]. As Luke et al. described the ORR of only 5.5% at a median follow-up time of 50.3 weeks and OS of 9.6 months [14]. Because MUM patients either are excluded from large randomized trials or represent a very small minority of patients in these trials [7], there is no prospectively proven therapy for MUM. The current treatment paradigm for this disease is based on the recommendations for metastatic cutaneous melanoma. However, there are local interventions as well such as chemoembolization or high-dose chemotherapy liver infusions that further guide therapies for MUM [1]. Unlike some of the cutaneous melanomas, uveal melanoma does not harbor BRAF mutations that are the targets of currently available therapies such as vemurafenib or dabrafenib [1].

Chemotherapeutic agents have been used without any significant response. Dacarbazine has shown a limited response in MUM. In SUMIT trial, the ORR was 0% in placebo+dacarbazine arm [21]. Other chemotherapeutic agents used in MUM are cisplatin, treosulfan, fotemustine and temozolomide with similar results [22, 23]. As mentioned above, selumetinib that is an MEK 1/2 inhibitor has been granted an orphan status by FDA due to better ORR (14%) compared to temozolomide or dacarbazine [9]. In this phase II trial, among 101 treatment-naïve or pre-treated MUM patients, the median progression-free survival (PFS) was significantly improved to 15.9 weeks vs. 7 weeks in selumetinib compared to chemotherapy respectively and the OS was increased to 11.8 months vs. 9.1 months in selumetinib compared to chemotherapy respectively [10]. Following these promising results, SUMIT trial, NCT01974752 was designed comparing selumetinib in combination with dacarbazine in systemic treatment-naïve MUM. However, this trial failed to meet its primary endpoints. Median PFS in selumeinib + dacarbazine arm was not significantly different from dacarbazine + placebo arm (2.8 vs. 1.8 months; HR 0.78(95% CL 0.48–1.27) [21].

Immune checkpoint inhibitors have been used in MUM in various cases as well [11–16]. Anti-CTLA4, ipilimumab is one of the first and most frequently used immune checkpoint inhibitor in MUM but has shown the response rate of only 5–10% and OS of 6.0–9.7 months [12, 14, 20, 24–26]. Long-term survivor was alive at 140+ weeks with PR to ipilimumab after receiving ten cycles of ipilimumab. He had received selumetinib, pegylated arginine deiminase before the initiation of ipilimumab and had a delayed progression [14]. Rodriguez et al. conducted the first phase II trial (GEM-1 trial) on MUM and reported PR in only one (7.7%) of thirteen evaluable patients and SD in six (46.2%) patients at a median follow-up time of 5.5 months [26]. Zimmer et al. conducted a phase II DeCOG trial on pre-treated and treatment-naïve MUM patients and reported median PFS of only 2.8 months and median OS of only 6.8 months [27]. Anti-PD-1 nivolumab and pembrolizumab have also been used in MUM in various instances without any promising results as the activity of PD-1 inhibition in uveal melanoma is not well described yet. Kottschade L.A. et al. treated ten patients with pembrolizumab who were pre-treated with ipilimumab. Median PFS was 18 weeks. Of eight evaluable patients, one patient showed CR, two with PR and one patient showed SD. This patient demonstrating CR initially progressed after three cycles of ipilimumab. She was subsequently started on pembrolizumab and achieved CR after four cycles. The patient continued to exhibit CR for 49 weeks at the closure of the study [28]. The combination of ipilimumab and nivolumab have been approved by FDA for metastatic melanoma patients and have shown improved activity compared to single agents in clinical trials for metastatic cutaneous melanoma [29]. Heppt et al. reviewed MUM patients treated with PD-1 inhibitors alone or with a combination of anti-PD-1/ anti-CTLA4. They showed a confirmed response rate of 4.7% from anti-PD-1 alone. Fifteen patients were treated with ipilimumab/nivolumab, and PR was observed in two patients only. One of these two patients received three cycles of ipilimumab + pembrolizumab. No prior ipilimumab only therapy was administered in this patient. The other patient with PR received eight cycles of ipilimumab/nivolumab before achieving PR. This patient also received no prior immunotherapy. These two cases were confirmed by the central review of the CT scan [30]. Karydis et al. performed a retrospective analysis on twenty-five patients pre-treated with ipilimumab receiving pembrolizuab. Only two patients achieved PR and six patients achieved SD. They reported a significant trend for improved outcomes in patients with extrahepatic disease contrary to our patient who had hepatic disease progression [31]. Chan et al. reported a case of MUM who experienced delayed progression 6 years after successful treatment of his choroidal melanoma and was treated with four cycles of ipilimumab/nivolumab after progression. The patient achieved PR and continue to have PR 10 months since the start of combination therapy (visible on CT scan from the baseline). Although subsequent immunotherapy was stopped 3 months after the initiation due to development of autoimmune hepatitis, uveitis, and diabetes [32].

Immune checkpoint inhibitors have the potential for adverse events and toxicities. Immunotherapy-related toxicity profile is also known as immune-related adverse events (irAEs). The typical mechanism of these irAEs is the enhancement of the autoimmunity because of these checkpoint inhibitors [33]. The frequency of irAEs is less in anti-PD-1 compared to anti-CTLA-4. However, the combination of anti-PD-1 and anti-CTLA-4 increases the incidence of these adverse events compared to any of these therapies alone. In the CheckMate 067 phase III trial, 55% patients experienced irAEs due to combination therapy vs. 16% patients receiving nivolumab and 27% patients receiving ipilimumab [29].

Hepatotoxicity can be seen by both anti-PD-1 and anti-CTLA-4 therapy. Ipilimumab associated hepatotoxicity is reported in 2–9% cases and nivolumab associated hepatotoxicity is reported in 4% cases [29, 34]. Hepatotoxicity is more common with ipilimumab/nivolumab combination. Up to 20% patients have been reported to experience grade 3 or greater hepatotoxicity [35]. Typically, hepatotoxicity results in elevation of aspartate aminotransferase (AST) and/or alanine aminotransferase (ALT) but can result in elevation of bilirubin as well in advance cases. Most of the patients experiencing hepatotoxicity are asymptomatic but can present with fever and fatigue. Typical time of onset for hepatotoxicity is 8–12 weeks but can also be seen several months later [36]. Management of hepatotoxicity depends on the grade. Grade 2 hepatotoxicity results in AST and/or ALT elevations > 2.5 times upper normal limit (UNL) but < 5-time UNL. Management of grade 2 toxicity is through withholding the checkpoint inhibitors and monitoring of the liver function tests till resolution. Grade 3 or greater hepatotoxicity is the elevation of AST and/or ALT > 5-times UNL or > 3-times UNL. Management is through stopping the immunotherapy permanently and initiation of high-dose corticosteroids (prednisone 1–2 mg/kg/day or equivalent). Gradual tapering is considered once symptoms subside to grade 1. Sometimes the long-term use of low-dose corticosteroids is needed due to relapse of the symptoms as happened with our patient [37]. In patients refractory to corticosteroids, other treatments such as mycophenolate mofetil (500 mg every 12 h), anti-thymocyte globulin therapy have been reported as well [38]. Studies have shown that the immunosuppressive therapy does not interfere with the efficacy of the immune checkpoint inhibitors [37, 39, 40].

Although, irAEs are associated with significant morbidity and warrant immediate intervention, interestingly, studies have shown that there exists a correlation between the incidence of irAEs and treatment-related outcomes [41–43]. A recent prospective analysis of 290 patients at MD Anderson showed that the patients with grade ≥ 3 irAEs had significantly improved ORR compared to the patients with grade < 3 irAEs (25% vs. 6%; *P* = 0.039). Further, the patients with grade ≥ 3 irAEs had a longer median time to progression (30 weeks vs. 10 weeks, *p* = 0.0040) [42]. In another retrospective study of 148 malignant melanoma patients treated with nivolumab, a statistically significant difference in OS was reported among patients with any grade of irAE compared to the patients without any irAEs (P ≤ 0.001) [43]. Our patient’s clinical course relates well with these reported studies as shown by great objective response observed after experiencing high-grade transaminitis. However, we have not identified any study relating autoimmune hepatitis and the treatment response in patients with malignant melanoma treated with immune checkpoint inhibitors.

## Conclusion

This is the unique report of a case of a MUM patient treated with combination anti-PD-1/anti-CTLA-4 therapy showing a durable response despite possessing worse prognostic features (GNA11 mutation, older age at presentation, male gender, short metastasis free interval and extraocular extension ciliary body involvement). Our patient has achieved a durable response to the combination therapy despite early progression from the original ocular melanoma treatment and has continued to do well 22 months after four cycles of ipilimumab / nivolumab followed by one dose of nivolumab without any evidence of the recurrent disease. Although restaging MRI scans continued to some evolutionary changes in previously described lesions, restaging PET/CT scans showed no suspicious metabolic activity. A latest abdominal MRI scan (22 months post-treatment) showed further retraction of the liver lesions. As mentioned above, few patients have demonstrated PR to monotherapy with immune checkpoint inhibitors, but the response rate is very low (2.6–5.7%) [14, 26]. However, few case reports and series have demonstrated a favorable response to the immune checkpoint inhibitors combination therapy [28, 30]. And these responses were seen in patients who had delayed progression from the initial ocular melanoma treatment. This approach may represent a viable option for MUM patients not responsive to single agent anti-PD-1 therapy as this combination is now approved for metastatic melanoma [28], and has shown improvement in the response rates and duration of response. However, it will be interesting to know the results of clinical trials of this combination therapy (clinicaltrials.gov NCT01585194 and NCT02626962) in MUM patients. Their findings may confirm that these observations are due to the combination therapies. This will be a paradigm shift in clinician’s approach towards MUM patients. Finally, it is important to consider that the combination therapy does increase the risk of irAEs and the patients should be carefully monitored for such adverse events.
